# Neuroprotective Effects of Ceftriaxone Involve the Reduction of Aβ Burden and Neuroinflammatory Response in a Mouse Model of Alzheimer’s Disease

**DOI:** 10.3389/fnins.2021.736786

**Published:** 2021-09-29

**Authors:** Maria A. Tikhonova, Tamara G. Amstislavskaya, Ying-Jui Ho, Anna A. Akopyan, Michael V. Tenditnik, Marina V. Ovsyukova, Alim A. Bashirzade, Nina I. Dubrovina, Lyubomir I. Aftanas

**Affiliations:** ^1^Laboratory of the Experimental Models of Neurodegenerative Processes, Department of Experimental Neuroscience, Scientific Research Institute of Neurosciences and Medicine (SRINM), Novosibirsk, Russia; ^2^Laboratory of Translational Biopsychiatry, Department of Experimental Neuroscience, Scientific Research Institute of Neurosciences and Medicine (SRINM), Novosibirsk, Russia; ^3^Department of Neuroscience, Institute of Medicine and Psychology, Novosibirsk State University, Novosibirsk, Russia; ^4^Department of Psychology, Chung Shan Medical University Hospital, Chung Shan Medical University, Taichung, Taiwan; ^5^Faculty of Life Sciences, Novosibirsk State University, Novosibirsk, Russia; ^6^Department of Clinical Neuroscience, Behavior and Neurotechnologies, Scientific Research Institute of Neurosciences and Medicine (SRINM), Novosibirsk, Russia

**Keywords:** neurodegeneration, drug repurposing, amyloid, microglia, cognition, behavior

## Abstract

Ceftriaxone (CEF) is a safe and multipotent antimicrobial agent that possesses neuroprotective properties. Earlier, we revealed the restoration of cognitive function in OXYS rats with signs of Alzheimer’s disease (AD)-like pathology by CEF along with its modulating the expression of genes related to the system of amyloid beta (Aβ) metabolism in the brain. The aim of this study was to determine the effects of CEF on behavior, Aβ deposition, and associated neuroinflammation using another model of an early AD-like pathology induced by Aβ. Mice were injected bilaterally i.c.v. with Aβ fragment 25–35 to produce the AD model, while the CEF treatment (100 mg/kg/day, i.p., 36 days) started the next day after the surgery. The open field test, *T*-maze, Barnes test, IntelliCage, and passive avoidance test were used for behavioral phenotyping. Neuronal density, amyloid accumulation, and the expression of neuroinflammatory markers were measured in the frontal cortex and hippocampus. CEF exhibited beneficial effects on some cognitive features impaired by Aβ neurotoxicity including complete restoration of the fear-induced memory and learning in the passive avoidance test and improved place learning in the IntelliCage. CEF significantly attenuated amyloid deposition and neuroinflammatory response. Thus, CEF could be positioned as a potent multipurpose drug as it simultaneously targets proteostasis network and neuroinflammation, as well as glutamate excitotoxicity, oxidative pathways, and neurotrophic function as reported earlier. Together with previous reports on the positive effects of CEF in AD models, the results confirm the potential of CEF as a promising treatment against cognitive decline from the early stages of AD progression.

## Introduction

Drug repurposing (also called drug repositioning or drug reprofiling) is a process of redeveloping a compound for application in a different pathology and finding new therapeutic indications for the existing drugs. The premise of repositioning is that the drugs that have previously passed clinical trials will minimize the risk of failure in future late-stage clinical trials due to toxicity and thus lead to faster drug approvals (Li and Jones, 2012). It has been growing in importance in the last few years and becoming mainstream in the drug research area and industry. This strategy appeared to be quite an effective approach in psychopharmacology as well. For instance, the antibiotic minocycline was proposed as an effective adjuvant treatment of schizophrenia to improve its negative symptoms (Zhang and Zhao, 2014). The present study was focused on another antimicrobial drug with neuroprotective properties, ceftriaxone (CEF).

CEF is a cephalosporin antibiotic drug of the 3rd generation. It is highly soluble in water and penetrates the blood–brain barrier (Nau et al., 1993). Pathological accumulation of the glutamate, the main excitatory neurotransmitter, in synapses leads to excitotoxic death of neurons in a number of neurological diseases. Glutamate is eliminated from the synaptic cleft mainly by means of glutamate transporter-1 (GLT-1). In 2005, it was hypothesized that CEF might be effective for the treatment of several neurodegenerative disorders associated with elevated glutamate levels, including cerebral ischemia, amyotrophic lateral sclerosis, and epilepsy. This assumption was based on the ability of CEF to increase the activity of GLT-1 in astrocytes with subsequent normalization of glutamate levels. This property of CEF was revealed during a wide screening of more than 1,000 drugs on sections of organotypic cultures of rat spinal cord cells (Rothstein et al., 2005). In a number of further works, its anti-excitotoxic effect was confirmed (Chu et al., 2007; Lipski et al., 2007; Hota et al., 2008).

The beneficial effect of CEF has been demonstrated on motor deficits in rats in an experimental model of Parkinson’s disease (Leung et al., 2012). The drug is actively studied in preclinical studies on models of neurological disorders (ischemia, myotonic dystrophy, alcohol, and drug addiction, etc.) in animals (Hakami and Sari, 2017; Hammad et al., 2017; Krzyzanowska et al., 2017; LaCrosse et al., 2017; Sicot et al., 2017; Stennett et al., 2017). Materials appeared on clinical trials of the CEF as a neuroprotective agent in amyotrophic lateral sclerosis (Cudkowicz et al., 2014) or Parkinson’s disease dementia.^1^ As glutamate-induced excitotoxicity is a prominent event in AD brains, the effects of the CEF in AD models were examined as well. The studies revealed positive effects of the CEF on AD-like pathology (Zumkehr et al., 2015; Hefendehl et al., 2016; Tikhonova et al., 2017). However, the additional mechanisms of its neuroprotective effects such as an activity targeted at proteostasis network or pathological aggregation of proteins were suspected.

Pathological aggregation and accumulation of Aβ and associated neuroinflammation in the brain tissue is considered to play a core role in the pathogenesis of AD (Selkoe and Hardy, 2016). Early stages of AD are associated with disturbances in amyloid metabolism and accumulation of amyloid oligomers that are the most toxic forms of amyloid that lead to synaptic and neuronal dysfunctions and initiate the pathological cascade (Haass and Selkoe, 2007; Mroczko et al., 2018). However, the potential impact of the CEF on this mechanism is scantily studied.

In recent years, when studying the mechanisms of the neuroprotective action of the CEF in models of neurodegenerative diseases (Alexander’s disease, Parkinson’s disease) *in vitro*, its ability to directly influence the expression and pathological aggregation of proteins that cause neurotoxicity and neurodegeneration was found (Bachetti et al., 2010; Ruzza et al., 2014). Our group revealed the CEF modulating the expression of genes related to the system of Aβ metabolism in the brain of 5-month-old OXYS rats in a model of an early stage of AD-like progression (Tikhonova et al., 2018). Here we checked whether CEF might influence Aβ burden and associated neuroinflammatory response in the brain at early stages of AD-like pathology. The aim of this study was to determine the effects of the CEF on behavior, Aβ deposition, and neuroinflammation using another model of an early AD-like pathology induced by Aβ neurotoxicity in mice.

## Materials and Methods

### Reagents

The following main reagents were used: Aβ25–35 fragment (Sigma, United States), CEF (Roche, Switzerland), a mouse monoclonal antibody to Aβ (cat. # NBP2-13075, 1:1,000 dilution; Novus Biologicals, United States), a rat monoclonal antibody to CD54/ICAM-1 (cat. # 16-0542-81, 1:300 dilution; eBioscience, Thermo Fisher Scientific, United States), a goat polyclonal antibody to microglial marker AIF-1/IBA1 (cat. # NB100-1028, 1:200 dilution; Novus Biologicals, United States), an Alexa Fluor 568-conjugated goat anti-mouse IgG polyclonal antibody (cat. # ab175473, 1:400 dilution, Abcam, United Kingdom), an Alexa Fluor 594-conjugated goat anti-rat IgG polyclonal antibody (cat. # ab150160, 1:500 dilution; Abcam, United Kingdom), and an Alexa Fluor 488-conjugated donkey anti-goat IgG polyclonal antibody (cat. # ab150129, 1:200 dilution; Abcam, United Kingdom).

### Experimental Procedures Involving Animals

Male C57Bl/6J mice (2 months old, 20–25 g) from the Federal State Budgetary Scientific Institution “Scientific Research Institute of Neurosciences and Medicine” (SRINM) (Novosibirsk, Russia) were used. Animals were kept on a standard laboratory diet and under standard conditions (light–dark cycle: 14 h light and 10 h dark; temperature: 20–22°C; relative humidity: 50–60%). All the experimental procedures were carried out in accordance with the guidelines of the NIH Guide for the Care and Use of Laboratory Animals and were approved by the Institutional Animal Care and Use Committee of the SRINM. Every effort was made to minimize the number of animals used and their suffering.

Experiments were conducted on a pharmacological model of neurodegeneration caused by central injection of an amyloid beta (Aβ) fragment 25–35. Mice were subdivided into four groups (15–20 animals each): (1) bilateral injections of sterile water into the lateral ventricles of the brain (i.c.v.) and intraperitoneal (i.p.) administration of saline (0.9% NaCl solution, 100 μl/10 g) for 36 days, (2) bilateral i.c.v. injections of sterile water and i.p. administration of CEF (100 mg/kg/day for 36 days), (3) bilateral i.c.v. injections of an Aβ fragment (Aβ25–35) and i.p. administration of saline for 36 days, and (4) bilateral i.c.v. injection of Aβ25–35 and i.p. administration of CEF for 36 days. All animals underwent stereotaxic surgery on day 0. The treatment with CEF was started on day 1. During the 2nd–5th weeks after the introduction of Aβ or vehicle into cerebral ventricles, behavioral testing was performed, after which biological samples were collected. On day 37, four randomly selected mice per group were sacrificed by exposure to CO_2_ and transcardially perfused with phosphate-buffered saline (PBS) and followed by 4% paraformaldehyde in PBS; then, their brains were rapidly removed and post-fixed in PBS containing 30% sucrose at 4°C. After being immersed in the embedding Tissue-Tek O.C.T. compound (Sakura Finetek, United States), the brains were frozen and stored at −70°C until sectioning into 30-μm-thick slices with a cryostat HistoSafe MicroCut-SADV (China).

#### The Model of Alzheimer’s Disease and Drug Administration

Aβ25–35 was dissolved in sterile water at a concentration of 1 mg/ml and stored at −20°C until use. Before administration to the animals, the prepared Aβ solution was thawed and incubated for 4 days at 37°C to form aggregates. Injections into cerebral ventricles were performed as described earlier (Tikhonova et al., 2020). The mice were anesthetized by administration of a 2.5% solution of avertin (2,2,2-tribromoethanol and 2-methyl-2-butanol, 100 μl/10 g, i.p.; Sigma-Aldrich Co.). The Aβ solution or sterile water was injected bilaterally with a Hamilton syringe (25 μl, model 1702 RN SYR, with a 22s ga needle, 2 in.), using a micropump (injection rate 0.8 μl/min). The needle was left at the injection site for 2 min after the injection. A total of 10 μl of the solution (9.4 nmol) were injected. The following coordinates adapted from the mouse brain atlas were used (Paxinos and Franklin, 2013): AP: -0.5 mm, ML: ±1 mm, and DV: −3 mm from the bregma, midline, and skull surface, respectively.

The rationale behind the CEF dosage (100 mg/kg/day) adopted in the current study was based on our recent studies showing neuroprotective effects of CEF in correcting behavioral and neuronal deficits (Tikhonova et al., 2017) and modifying the expression of genes related to the system of Aβ metabolism in the brain (Tikhonova et al., 2018) in another AD model (OXYS rats). Mice were weighed weekly during the experiment to adjust the drug dosage. The drug administration that preceded the testing of behavior or the collection of bio-samples was performed in 1 day prior to the corresponding manipulation in order to avoid the acute effects of CEF.

### Behavioral Tests

Each animal was handled for 5 min/day on three consecutive days, before taking into the experiment.

#### IntelliCage

7–8 mice of each group were tested in an observer-independent setting using the IntelliCage apparatuses (TSE systems, Germany). The IntelliCage for mice with minor modifications has been described in sufficient details in a number of studies (Galsworthy et al., 2005; Barlind et al., 2010; Benner et al., 2015; Fischer et al., 2017). Briefly, it consists of a transparent cage (20.5 × 55 × 37.5 cm; Tecniplast, 2000P) equipped with four operant learning chambers (15 × 15 × 21 cm), which fit in to the corners of the housing cage. Each corner chamber holds two bottles of water (eight bottles per cage in total) that are separated from the living part of the cage by a circular automatically closable door (13 mm in diameter) with sensors for controlling access to bottles and an RFID antenna for identifying mice. Individual identification of a mouse was provided by subcutaneous implantation of a microchip into its interscapular region under light anesthesia 1 week before the experiment. To study the advanced conditioned responses (i.e., patrolling behavior), there are three colored LEDs above each door. Doors open when a sensor is activated with a mouse’s nosepoke. Having opened the door, the mouse receives positive reinforcement (drinking water) or negative stimulation (trigger an air puff). IntelliCage Plus software controls an experimental protocol and registers automatically the number and duration of visits to corners, nosepokes, and licks. As a social group containing up to 16 mice could be tested at a time in the IntelliCage, we used two IntelliCage devices, one for Aβ-treated mice and another for vehicle (H_2_O)-injected mice. A protocol in the experiment included the tests for place learning, place learning reversal, àvoidance conditioning, avoidance extinction, and patrolling behavior. The protocol details are presented as Supplementary Table 1.

The rest of the mice were tested in the *T*-maze, Barnes, open field, and passive avoidance tests. They were housed in groups of four to five in acrylic cages (25 × 40 × 20 cm) in an animal room. In 2 weeks after surgery (i.c.v. Aβ administration, day 0), the mice were subjected to tests for behavioral phenotyping: the *T*-maze test on days 15–17, Barnes test on days 20–25, open field on day 27, and passive avoidance test on days 34–36. All observations were performed during the light phase between 12:00 and 20:00 h. For behavioral testing, the animals were placed individually in a clean cage (25 × 40 × 20 cm) and transported to a dim observation room (28 lx of the red light) with sound isolation reinforced by a masking white noise of 70 dB. Performance in the behavioral tests was monitored using a video camera (Sony, China) positioned above an apparatus and processed with original EthoVision XT software (Noldus, Netherlands). The test equipment was cleaned using 20% ethanol and thoroughly dried before each test trial.

#### The *T*-Maze Test

The test was conducted according to the spontaneous alteration protocol at red lighting of 28 lx (Deacon and Rawlins, 2006). *T*-shaped apparatus consists of a start arm (30 × 7 cm) and two side arms (37 × 7 cm) with plastic walls of 20 cm high. Start zone in the start arm is 18 × 7 cm, while central zone between the side arms is 7 × 7 cm. All compartments are separated by automatic slide doors controlled remotely by the EthoVision XT software (Noldus, Netherlands). The test consisted of three trials per day during three consecutive days for each mouse. Each trial included two choice runs. At the beginning of each run, a mouse was placed in the start zone. During each run, the mouse made a choice of a side arm by entering into it. In the first run, right after the choice was made, a slide door separating the side arm with the mouse shut down, and the mouse had stayed in the selected arm for 30 s until the second run. In the second run, a mouse should choose a side arm opposite to that chosen in the first run (correct choice). Correct responses in the nine trials were recorded. The percentage of correct choices was regarded as an index of working memory (Deacon and Rawlins, 2006; Paul et al., 2009). The duration of each run was restricted by 90 s.

#### Barnes Maze Test

The test assesses spatial learning and memory. A mouse was placed on an elevated open circular arena (*d* = 120 cm, height from the floor = 90 cm) with 40 holes (*d* = 5 cm, distance between holes = 8 cm). An escape box was placed beneath one of the holes, and its location was randomly assigned to four positions for each mouse. Aversive bright lighting (1,000 lx) and the stress of being in the open space motivated an animal to search for the escape box to hide. Visual cues placed in the testing room provided spatial orientation. Testing was conducted according to the standard protocol (Dudchenko, 2004; Paul et al., 2009) and consisted of three phases: habituation (1 day, two sessions of 3 min), acquisition (4 days, four sessions of 3 min/day), and testing trial (1 day, one session of 60 s). *Habituation*: a mouse was placed near the hole with the escape box attached (“goal hole”); if the animal did not find the goal hole within 3 min, it was gently guided to the escape box and left there for 60 s. *Acquisition*: the animal was placed in the center of a platform and was free to explore the platform and search for the goal hole and escape box; if the animal did not find the goal hole within 3 min, it was gently guided to the escape box and left there for 60 s. The latency of finding the goal hole was recorded. Episodic memory was assessed as the dynamics of the latency in the four consecutive sessions on the first training day. During the *Testing trial*, the escape box was removed, and mice moved freely for 60 s. Exploratory activity (by the total number of nosepokes) and long-term memory and learning (by the percentage of the nosepokes to the goal hole) were evaluated.

#### The Open Field Test

This test was carried out in an apparatus with a square arena (40 × 40 cm) and plastic walls 37.5 cm high brightly lit from above (1,000 lx). A mouse was placed near the wall, and its movements were recorded for 10 min. The following parameters were determined: general locomotion (the distance traveled in cm), vertical locomotor and exploratory activity (rearing number), anxiety (time spent in the central part of the arena), and emotionality (defecation number).

#### The Passive Avoidance Test

Training on the passive avoidance reaction was performed by a standard single-session method in an experimental chamber with dark and light compartments and an automated Gemini Avoidance System apparatus (San Diego Instruments, CA, United States) as described in detail earlier (Tikhonova et al., 2020). The Gemini software automatically recorded the latency of the transfer to the dark compartment, and the data of testing served as a measure of acquisition of the conditioned passive avoidance reaction.

### Nissl Staining and Immunohistochemical Analysis

#### Nissl Staining

Coronal slices along the frontal cortex (*AP* = 2.93–2.57 mm) or hippocampus (*AP* = -1.91 to −2.45 mm) of each mouse brain were made. Unstained brain sections were identified according to the mouse brain atlas (Paxinos and Franklin, 2013). Nissl staining, used to measure the neuronal density, was performed as described in our previous reports (Weng et al., 2016; Tikhonova et al., 2017). The image was captured and analyzed using a microscope Nikon Eclipse Ci (Nikon, China) coupled to a Nikon DS-Fi2 camera (Nikon, China) and Image Pro Plus Software 6.0 (Media Cybernetics, CA, United States). The neuronal density was measured by a semi-quantitative method as described earlier (Ho et al., 2014; Tikhonova et al., 2017) since it is difficult to directly count the number of neurons in a 30-μm thick brain section because the neurons are tightly packed. We calculated the percentage of an area of interest (AOI) in the 3rd layer of the frontal cortex (AOI size: 93,023 μm^2^) and CA1 or CA3 area of the hippocampus (AOI size: 88,502 μm^2^) occupied by Nissl-stained cells. The analyzer was blind to the treatment.

#### Immunohistochemical Analysis

Brain sections for the Immunohistochemical (IHC) analysis were randomly taken from the same animals that were used for the histological assay counting the density of neurons with Nissl staining. The IHC analysis was performed according to a protocol described in detail previously (Tikhonova et al., 2017, 2020). Antibodies used are listed in the Reagents section. The fluorescence images were finally obtained by an Axioplan 2 (Carl Zeiss) imaging microscope and then analyzed in Image Pro Plus Software 6.0 (Media Cybernetics, MD, United States). Fluorescence intensity associated with the expression of specific proteins (Aβ, CD54, or IBA1) was measured as background-corrected optical density (OD) with subtraction of staining signals of the non-immunoreactive regions in the images converted to grayscale. The AOI size was 18,208 μm^2^ in the 3rd layer of the frontal cortex or in the dentate gyrus of the hippocampus, 19,353 μm^2^ in the CA1 area, or 26,100 μm^2^ in the CA3 area of the hippocampus. The analyzer was blind to the treatment.

### Data Analysis

The results were presented as mean ± SEM and compared using a two-way ANOVA followed by *post hoc* Fisher’s least significant difference (LSD) test. The independent variables for the two-way ANOVA were Aβ administration [control (mice administered i.c.v. with H_2_O) or Aβ-treated mice] and CEF treatment (saline- or CEF-treated mice). Repeated-measures ANOVA followed by Fisher LSD *post hoc* comparison was applied to analyze the data of the passive avoidance test/Barnes test with Aβ administration and CEF treatment as between-subject variables and time (training or test/number of a session on the first day of training) as a repeated measure. The level of significance was defined as *p* < 0.05. STATISTICA 10.0 software (StatSoft, Tulsa, OK, United States) was used to perform all statistical analyses.

## Results

### Analysis of Behavioral Effects of Ceftriaxone in the Aβ-Induced Mouse Alzheimer’s Disease Model

**FIGURE 1:**
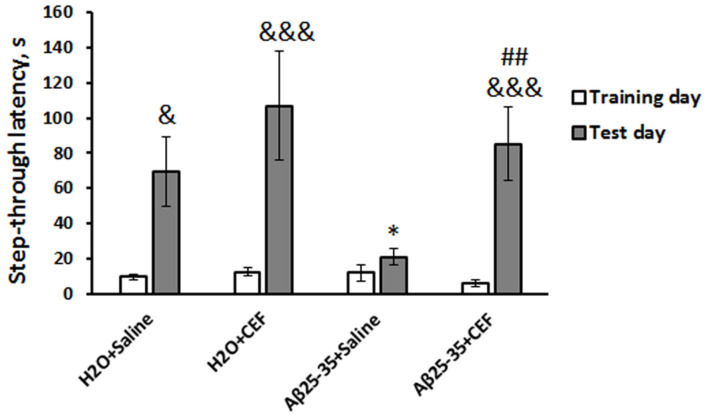
Effects of the CEF and Aβ25–35 administration (AD model) on memory retrieval in mice in the passive avoidance test. The data are expressed as mean ± SEM of the values obtained in an independent group of animals (*n* = 5–6 per group). Statistically significant differences: ^&^*p* < 0.05, ^&&&^*p* < 0.001 compared with values of the same group on the training day; ^∗^*p* < 0.05 vs. the “H_2_O + saline” group on the test day; ^##^*p* < 0.01 vs. the “Aβ + saline” group on the test day.

**FIGURE 2:**
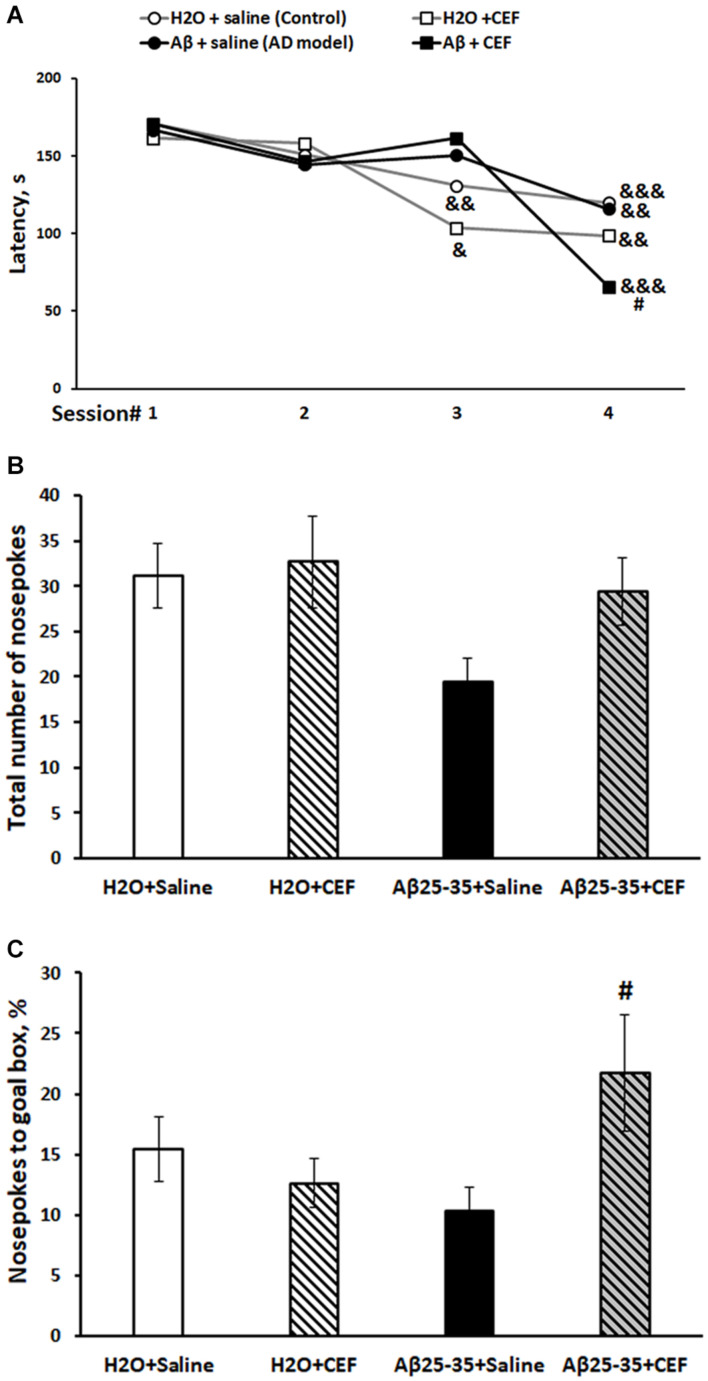
Effects of the CEF and Aβ25–35 administration (AD model) on spatial memory and learning in mice in the Barnes test. **(A)** Episodic memory and learning were evaluated by the latency (s) to find an escape box during the first day of training. **(B)** General exploratory activity was estimated by the total number of nosepokes on the test day. **(C)** Long-term spatial memory was evaluated by the percentage of nosepokes to the target hole on the test day. The data are expressed as mean ± SEM of the values obtained in an independent group of animals (*n* = 7–15 per group). Statistically significant differences: ^&^*p* < 0.05, ^&&^*p* < 0.01, ^&&&^*p* < 0.001 compared with values of the same group on the first training session; ^#^*p* < 0.05 compared with respective values of the “Aβ + saline” group.

**FIGURE 3:**
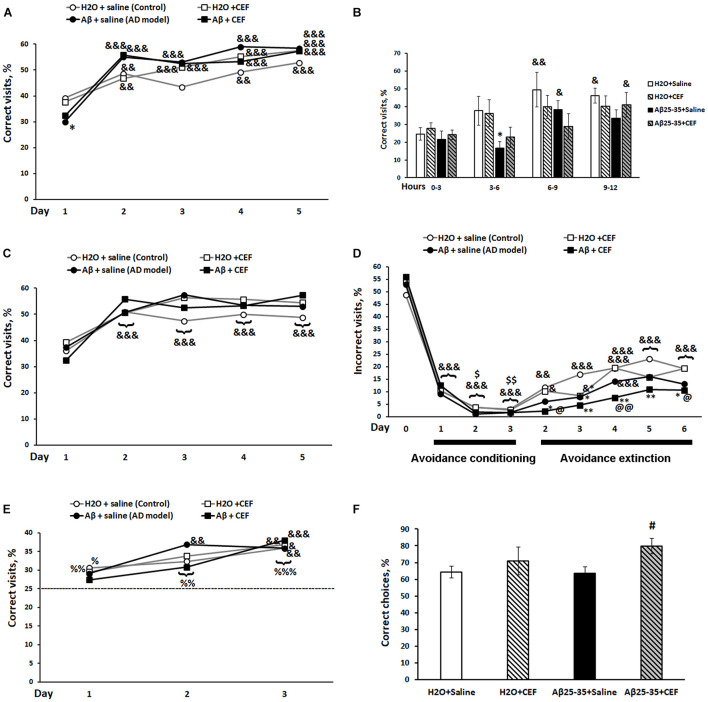
Effects of the CEF and Aβ25–35 administration (AD model) on the place learning **(A,B)**, place learning reversal **(C)**, avoidance conditioning and extinction **(D)**, patrolling behavior **(E)** in the IntelliCage, or on the working spatial memory in the *T*-maze test **(F)** in mice. The dynamics of place learning in the IntelliCage was evaluated during the whole period of the test with the values pooled daily **(A)** or during the first day of this phase of the test with the values pooled for 3 h **(B)**. The data are expressed as means **(A,C–E)** or mean ± SEM **(B,F)** of the values obtained in an independent group of animals (*n* = 7–8 per group). Statistically significant differences: ^&^*p* < 0.05, ^&^^&^*p* < 0.01, ^&^^&^^&^*p* < 0.001 compared with values of the same group at the first timepoint of the phase, for avoidance conditioning—compared with values of the same group on the last day of the previous phase (day 0 in the figure), for avoidance extinction—compared with values of the same group on the last day of the previous phase (day 3 of the avoidance conditioning test); ^$^*p* < 0.05, ^$$^*p* < 0.01 compared with values of the same group on the first day of the avoidance conditioning test; ^%^*p* < 0.05, ^%^^%^*p* < 0.01, ^%^^%^^%^*p* < 0.001 compared with the chance level (25%); ^∗^*p* < 0.05, ^∗∗^*p* < 0.01 compared with respective values of the “H_2_O + saline” group; ^#^*p* < 0.05 compared with respective values of the “Aβ + saline” group; ^@^*p* < 0.05, ^@^^@^*p* < 0.01 compared with respective values of the “H_2_O + CEF” group.

The efficacy of CEF in recovering cognitive function was evaluated using behavioral phenotyping: the test of passive avoidance learning (Figure 1), Barnes test (Figure 2), *T*-maze test (Figure 3F), and IntelliCage paradigm (Figures 3A–E). To assess the CEF effects on general locomotion and exploratory activity, the open field test was applied (Table 1).

**TABLE 1:** Effects of the CEF and Aβ25–35 administration (AD model) on the behavior of mice in the open field test.

Index	Group				Effects (*F*, *p*)
	
	H_2_O + saline	H_2_O + CEF	Aβ25–35 + saline	Aβ25–35 + CEF	
Locomotor activity (path length, cm)	2728.0 ± 106.2	3224.7 ± 223.2*	2668.2 ± 157.9	2580.5 ± 184.9$	**Aβ:** *F*(1,37) = 4.4, *p* < 0.05; **CEF:** *F*(1,37) = 1.5, *p* > 0.05; **Aβ × CEF:** *F*(1,37) = 3.0, *p* > 0.05
Exploratory activity (no. of rearings)	65.9 ± 3.9	63.5 ± 5.5	60.1 ± 5.3	58.4 ± 7.0	**Aβ:** *F*(1,37) < 1; **CEF:** *F*(1,37) < 1; **Aβ × CEF:** *F*(1,37) < 1
Anxiety (time in the center, s)	31.8 ± 4.1	34.1 ± 7.7	26.9 ± 3.7	21.9 ± 2.7	**Aβ:** *F*(1,37) = 3.1, *p* > 0.05; **CEF:** *F*(1,37) < 1; **Aβ × CEF:** *F*(1,37) < 1
Emotionality (no. of fecal boluses)	1.40 ± 0.48	1.17 ± 0.65	2.08 ± 0.59	2.14 ± 0.51	**Aβ:** *F*(1,37) = 1.8, *p* > 0.05; **CEF:** *F*(1,37) < 1; **Aβ × CEF:** *F*(1,37) < 1

Data are presented as mean ± SEM of the values obtained in an independent group of animals (*n* = 7–15 per group). Statistically significant differences: *p < 0.05 vs. the “H_2_O + saline” group; $ *p* < 0.05 vs. the “H_2_O + CEF” group.

#### The Passive Avoidance Test

There was a significant influence of the CEF treatment [*F*(1,17) = 5.2, *p* < 0.05], learning (repeated measures) [*F*(1,17) = 33.0, *p* < 0.001], and of the interaction between these factors [*F*(1,17) = 6.2, *p* < 0.05] on the step-through latency. Latency to enter a dark compartment during training (before the foot shock) did not differ significantly among the experimental groups (Figure 1). As evidence of learning on testing day, 24 h after receiving the foot shock, control mice of the H_2_O + saline group showed increased step-through latencies, often ∼10-fold greater than latencies on training day, so did the mice of the H_2_O + CEF and Aβ25–35 + CEF groups. In contrast to those groups, the step-through latencies of Aβ25–35 + saline-treated mice (AD model) were sharply reduced and did not differ from the latencies during training (*p* > 0.05), indicating memory impairment. Thus, CEF treatment prominently improved the learning deficit in the mouse Aβ25–35-induced AD model as evidenced by a significantly longer retention latencies in comparison with Aβ25–35 + saline-treated mice (*p* < 0.01). Noteworthy, CEF treatment augmented step-through latencies in the Aβ25–35 + CEF group up to values observed in control mice of the H_2_O + saline group.

#### Barnes Test

Episodic spatial memory was estimated in the Barnes test. Latencies of finding the goal box at four trials on the first day of training were assessed. The dynamics of learning is summarized in Figure 2A. There was a significant influence of the learning (repeated measures) [*F*(3,117) = 17.0, *p* < 0.001] and of the interaction between the factors of learning and Aβ25–35 administration [*F*(3,117) = 3.01, *p* < 0.05]. Control mice of the H_2_O + saline group (*p* < 0.01), as well as mice of the H_2_O + CEF (*p* < 0.05) group, showed a significant decrease in the latency of finding the goal hole by the third trial, while mice of both groups administered with Aβ25–35 demonstrated the significant latency reduction by the fourth trial. However, animals of the Aβ25–35 + CEF group had shorter latency in the fourth trial than those of the Aβ25–35-induced AD model without CEF treatment (*p* < 0.05). On the test day, no significant effects of the factors on the index of exploration (the total number of nosepokes to holes) were found (Figure 2B), while the parameter of spatial memory and learning (% of the goal hole nosepokes) was significantly influenced by the interaction between the factors of Aβ25–35 administration and CEF treatment [*F*(1,38) = 4.9, *p* < 0.05; Figure 2C]. The percentage of the goal hole nosepokes was markedly augmented in the Aβ25–35 + CEF group as compared with the Aβ25–35 + saline group (*p* < 0.05).

#### IntelliCage

In the place learning test, there was a significant influence of learning (repeated measures) [*F*(4,104) = 76.03, *p* < 0.001] and of its interaction with the factor of Aβ25–35 administration [*F*(4,104) = 7.29, *p* < 0.001] on the percentage of correct visits (Figure 3A). All experimental groups demonstrated a significant increase in the percentage of correct visits on days 2–5 of place learning testing compared with the first day (learning period) of this phase. LSD *post hoc* test revealed that on the first day of place learning testing, the percentage of correct visits was substantially reduced in the Aβ25–35 + saline group in comparison with the control mice of the H_2_O + saline group (*p* < 0.01) indicating episodic memory and learning disturbances in mice exposed to the neurotoxic effects of Aβ25–35, while mice of the Aβ25–35 + CEF group did not show a significant decrease in the parameter as compared with the groups that did not receive Aβ25–35 injections. However, on the next days, the percentage of correct visits increased in mice of the Aβ25–35 + saline group to the level of mice treated with H_2_O instead of Aβ25–35. The most profound difference in the percentage of correct visits between the mice treated with Aβ25–35 of the Aβ25–35 + saline group and those of the H_2_O + saline group was noted during the period of 3–6 h on the first day, while mice of the Aβ25–35 + CEF group did not show a significant decrease in the parameter as compared with the groups that did not receive Aβ25–35 injections (learning (repeated measures) factor [*F*(3,75) = 7.29, *p* < 0.001], Aβ25–35 administration [*F*(1,25) = 9.2, *p* < 0.01]; Figure 3B).

In the place learning reversal test, there was a significant influence of learning (repeated measures) [*F*(4,104) = 33.83, *p* < 0.001] on the percentage of correct visits, as well as on the percentage of incorrect visits [*F*(4,104) = 33.0, *p* < 0.001], while the effects of other factors or their interaction were insignificant. All experimental groups demonstrated a significant increase in the percentage of correct visits (Figure 3C) and simultaneous significant decrease in the percentage of incorrect visits (data not shown) on days 2–5 of place learning reversal testing compared with the first day (learning period) of this phase. No significant intergroup differences were found. Neither intergroup differences were observed on the first day [learning (repeated measures) factor [*F*(3,75) = 10.71, *p* < 0.001] for the percentage of correct visits and learning (repeated measures) factor [*F*(3,75) = 15.79, *p* < 0.001] for the percentage of incorrect visits; data not shown]. Thus, the reversal learning ability was observed in all the groups studied. Aβ25–35 administration or CEF treatment did not affect this feature significantly.

Similarly, in the avoidance conditioning test, there was a significant influence of learning (repeated measures) [*F*(3,78) = 624.2, *p* < 0.001] on the percentage of incorrect visits, while the effects of other factors or their interaction were insignificant. All experimental groups demonstrated a significant decrease in the percentage of incorrect visits (Figure 3D) since the first day of training compared with the percentage of the corner at previous phase (the last day of the place learning reversal test when the corner was assigned as correct and mice were not punished for its visiting). On days 2–3 of the avoidance conditioning test, further decrease in the percentage of incorrect visits was observed in all groups as compared with the first day of training. Thus, the learning ability at avoidance conditioning was observed in all the groups studied. Aβ25–35 administration or CEF treatment did not affect this feature significantly.

The avoidance extinction test revealed a significant influence of learning (repeated measures) [*F*(5,130) = 26.1, *p* < 0.001] and Aβ25–35 administration [*F*(1,26) = 10.55, *p* < 0.01] on the percentage of visits to the corner that was assigned as incorrect during the avoidance conditioning test, while the effects of other factors or their interaction were insignificant. All experimental groups demonstrated a gradual increase in the percentage of the visits during the avoidance extinction test (Figure 3D). Mice of the H_2_O + saline or H_2_O + CEF group had shown the increased percentage of the visits since the second day of the avoidance extinction phase compared with the last day of the avoidance conditioning test. However, mice treated with Aβ25–35 revealed a retarded extinction of avoidance learning. Mice of the Aβ25–35 + saline group had demonstrated a significant increase in the percentage of the visits since the fourth day, while mice of the Aβ25–35 + CEF group since the fifth day of the avoidance extinction test. Noteworthy, values of the parameter in the Aβ25–35 + CEF group were significantly lower than those in the H_2_O + saline or H_2_O + CEF group on all days of the avoidance extinction test (Figure 3D).

The test for patrolling behavior also revealed a significant effect of learning (repeated measures) on the percentage of correct visits [*F*(2,52) = 21.1, *p* < 0.001], while the effects of other factors or their interaction were insignificant. According to LSD *post hoc* test, all experimental groups demonstrated a significant increase in the percentage of correct visits on the third day of testing compared with the first day of training. All groups had shown a significantly increased level of correct visits as compared with the chance level (25%) since the second day of the test for patrolling behavior (Figure 3E). Thus, the working memory was not disturbed in all the groups studied. Aβ25–35 administration or CEF treatment did not affect this feature significantly. However, it should be noted that the Aβ25–35-treated groups did not differ significantly in the percentage of correct visits in comparison with the chance level on the first day of the test for patrolling behavior, while the H_2_O-treated groups had significantly augmented level of correct responses compared with the chance level on the first day of the test. That may indicate to the retarded learning of a new rule in the Aβ25–35-treated groups.

#### *T*-Maze Test

When comparing the indices of the working spatial memory in the *T*-maze test using the spontaneous alteration protocol, a significant influence of CEF treatment [*F*(1,34) = 5.2, *p* < 0.05] but not the Aβ25–35 injection factor [*F*(1,34) < 1] or interaction between the factors [*F*(1,34) < 1] on the percentage of correct choices was revealed. The percentage of correct choices in the Aβ25–35 + CEF group was higher than that in the Aβ25–35 + saline group (*p* < 0.05) (Figure 3F).

#### Open Field Test

Evaluation of general locomotor and exploratory activity and some other parameters was carried out by an open field test. The results are summarized in Table 1. There was a significant influence of Aβ25–35 administration [*F*(1,37) = 4.4, *p* < 0.05] on the locomotion (distance traveled), while the effects of CEF treatment or interaction between the factors were insignificant. However, mice of the Aβ25–35 + saline group did not differ significantly from those of the H_2_O + saline group or the Aβ25–35 + CEF group in the distance traveled. Moreover, the groups studied did not differ significantly in the indices of exploratory activity (number of rearings), anxiety (time spent in the center of the arena), or emotionality (number of fecal boluses) as well.

### Analysis of Ceftriaxone Effects on Neuronal Density, Aβ Accumulation, and Neuroinflammation in the Aβ-Induced Mouse Alzheimer’s Disease Model

#### Nissl Staining

We have not found significant differences in the neuronal density in the frontal cortex or hippocampal CA1 and CA3 regions between the groups. Neither Aβ25–35 administration nor CEF treatment affected this feature in C57Bl6/J mice significantly. The detailed results are presented as Supplementary Table 2.

At the same time, a pronounced influence of Aβ25–35 administration or CEF treatment on Aβ accumulation and neuroinflammatory features was revealed.

#### Aβ Staining

**FIGURE 4:**
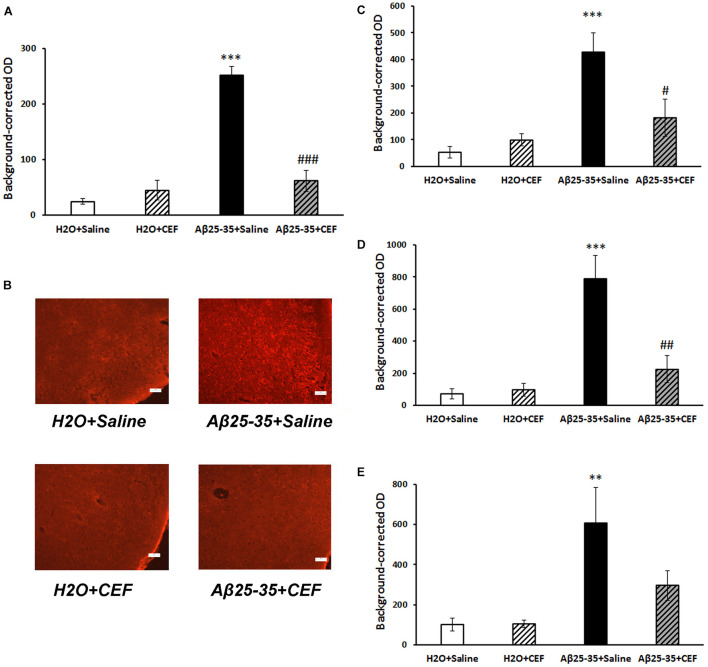
Effects of the CEF and Aβ25–35 administration (AD model) on the Aβ accumulation in the frontal cortex **(A,B)** or hippocampus (**C** in the CA1 area; **D** in the CA3 area; **E** in the dentate gyrus) in mice. **(A,C–E)** Quantitative results. The data are expressed as mean ± SEM of the values obtained in an independent group of animals (*n* = 3–4 per group). Statistically significant differences: ^∗∗^*p* < 0.01, ^∗∗∗^*p* < 0.001 vs. the “H_2_O + saline” group; ^#^*p* < 0.05, ^#^^#^*p* < 0.01, ^#^^#^^#^*p* < 0.001 vs. the “Aβ + saline” group. **(B)** Aβ immunoreactivity in the frontal cortex. Magnification, ×200; bar, 50 μm.

In mice subjected to central administration of the Aβ25–35, Aβ burden was significantly reduced after CEF therapy in the frontal cortex and hippocampus (Figure 4). Significant effects of the factors of Aβ25–35 administration [*F*(1,8) = 62.2, *p* < 0.001], CEF treatment [*F*(1,8) = 30.1, *p* < 0.001], and their interaction [*F*(1,8) = 46.3, *p* < 0.001] on the levels of Aβ in the frontal cortex in mice were found. Similarly, the content of Aβ was significantly augmented in the CA1 and CA3 regions or the dentate gyrus of the hippocampus in mice of the Aβ25–35 + saline group given Aβ25–35 injections compared with control mice of the H_2_O + saline group, and it decreased to the level of the control group after CEF therapy (Figures 4C,D,E). In the CA1 area of the hippocampus, significant effects of the Aβ25–35 administration [*F*(1,8) = 19.1, *p* < 0.01] and the interaction between the factors of Aβ25–35 and CEF treatment [*F*(1,8) = 7.8, *p* < 0.05] on the Aβ levels were observed. In the CA3 area of the hippocampus, significant effects of the Aβ25–35 administration [*F*(1,8) = 23.2, *p* < 0.01], CEF treatment [*F*(1,8) = 9.5, *p* < 0.05], and the interaction between the factors [*F*(1,8) = 11.3, *p* < 0.01] on the Aβ levels were found. Aβ25–35 administration [*F*(1,8) = 12.3, *p* < 0.01] but not CEF treatment or interaction of the factors influenced significantly the content of Aβ in the dentate gyrus of the hippocampus.

For neurodegenerative disorders and AD in particular, neuroinflammation is one of the key pathogenetic features. Hence, we evaluated the effects of CEF treatment on neuroinflammatory indices.

#### CD54 Expression

**FIGURE 5:**
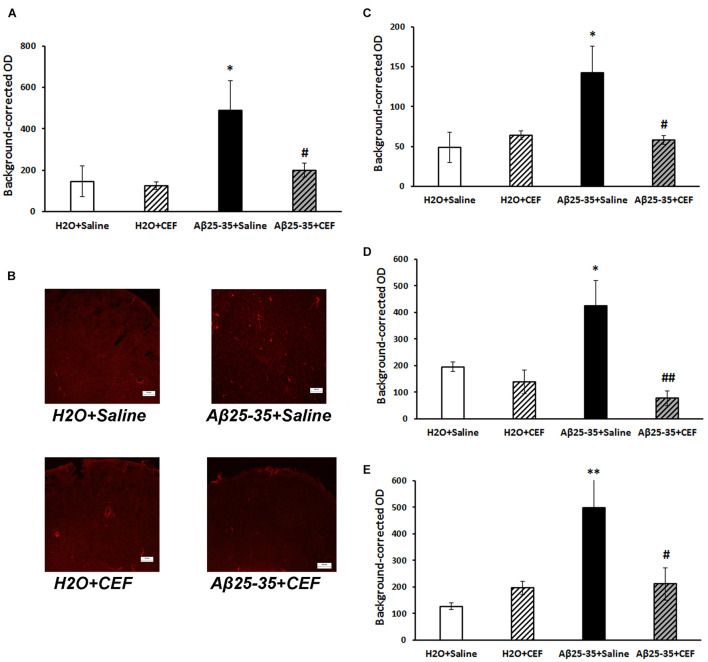
Effects of the CEF and Aβ25–35 administration (AD model) on the expression of inflammatory marker CD54 in the frontal cortex **(A,B)** or hippocampus (**C** in the CA1 area; **D** in the CA3 area; **E** in the dentate gyrus) in mice. **(A,C–E)** Quantitative results. The data are expressed as mean ± SEM of the values obtained in an independent group of animals (*n* = 3–4 per group). Statistically significant differences: ^∗^*p* < 0.05, ^∗∗^*p* < 0.01 vs. the “H_2_O + saline” group; ^#^*p* < 0.05, ^##^*p* < 0.01 vs. the “Aβ + saline” group. **(B)** CD54 immunoreactivity in the frontal cortex. Magnification, ×200; bar, 50 μm.

The expression of inflammatory marker CD54 was significantly increased in the frontal cortex and hippocampus in mice of the Aβ25–35 + saline group given Aβ25–35 injections compared with control mice of the H_2_O + saline group, while it decreased substantially to the level of the control group after CEF therapy (Figure 5).

Significant influence of Aβ25–35 administration [*F*(1,11) = 5.42, *p* < 0.05] was revealed on the levels of CD54 in the frontal cortex. In the CA1 area of the hippocampus, the interaction between the factors of Aβ25–35 and CEF treatment influenced significantly the expression of CD54 [*F*(1,10) = 5.5, *p* < 0.05]. In the CA3 area of the hippocampus, a significant effect of CEF treatment [*F*(1,9) = 9.5, *p* < 0.05] on the CD54 levels was found. Aβ25–35 administration [*F*(1,9) = 6.95, *p* < 0.05], as well as the interaction between the factors of Aβ25–35 and CEF treatment [*F*(1,9) = 5.85, *p* < 0.05], had a significant effect on the expression of CD54 in the dentate gyrus of the hippocampus.

#### Microglia Activation

**FIGURE 6:**
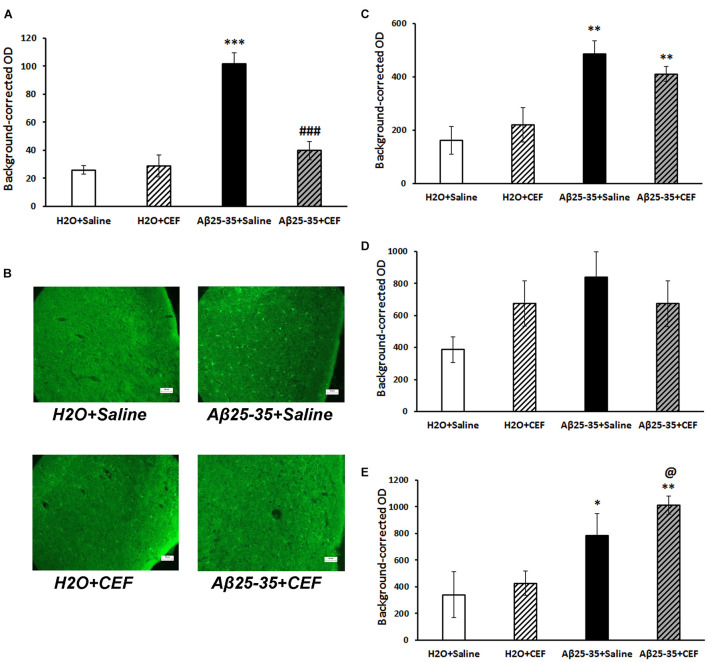
Effects of the CEF and Aβ25–35 administration (AD model) on the expression of microglial marker IBA1 in the frontal cortex **(A,B)** or hippocampus (**C** in the CA1 area; **D** in the CA3 area; **E** in the dentate gyrus) in mice. **(A,C–E)** Quantitative results. The data are expressed as mean ± SEM of the values obtained in an independent group of animals (*n* = 3–4 per group). Statistically significant differences: ^∗^*p* < 0.05, ^∗∗^*p* < 0.01, ^∗∗∗^*p* < 0.001 vs. the “H_2_O + saline” group; ^###^*p* < 0.001 vs. the “Aβ + saline” group; ^@^*p* < 0.05 vs. the “H_2_O + CEF” group. **(B)** IBA1 immunoreactivity in the frontal cortex. Magnification, ×200; bar, 50 μm.

Microglia activation was assessed by the expression of IBA1 marker. Its expression was significantly increased in the frontal cortex in mice of the Aβ25–35 + saline group given Aβ25–35 injections compared with control mice of the H_2_O + saline group (*p* < 0.001), while it decreased substantially to the level of the control group after CEF therapy (Figures 6A,B). Significant effects of the factors of Aβ25–35 administration [*F*(1,10) = 37.4, *p* < 0.001], CEF treatment [*F*(1,10) = 17.5, *p* < 0.01], and their interaction [*F*(1,10) = 21.0, *p* < 0.01] on the levels of IBA1 in the frontal cortex in mice were found.

At the same time, in the hippocampus, IBA1 expression was significantly influenced only by Aβ25–35 administration in the CA1 area [*F*(1,8) = 26.3, *p* < 0.001] or dentate gyrus [*F*(1,8) = 94.4, *p* < 0.001] but not in the CA3 region [*F*(1,8) = 2.8, *p* > 0.05], while the effects of CEF or interaction of the factors were insignificant (Figures 6C–E).

It should be noted that in the frontal cortex, Aβ burden had strong positive correlation with both CD54 expression (*r*_12_ = 0.74, *p* < 0.01) and microglia activation (*r*_12_ = 0.72, *p* < 0.01). Aβ accumulation also correlated positively with microglia activation in the hippocampal CA1 area (*r*_12_ = 0.82, *p* < 0.01) or dentate gyrus (*r*_12_ = 0.59, *p* < 0.05) but not in the CA3 region. Positive correlation between Aβ burden and CD54 expression was found in the hippocampal CA3 area (*r*_11_ = 0.61, *p* < 0.05) but not in CA1 or dentate gyrus.

## Discussion

Beta-lactam antibiotics including CEF have been considered an optimistic group of drugs for treating neurodegenerative disorders (Kumari and Deshmukh, 2021). Through modulating the transcription and expression of the GLT-1, CEF protects neurons from excitotoxic neuronal damage (Rothstein et al., 2005). Moreover, CEF ameliorates symptoms across multiple rodent models of neurological diseases and substance use disorders associated with glutamate excitotoxicity-induced neuronal dysfunction (Tai et al., 2019; Yimer et al., 2019; Smaga et al., 2020). Since the glutamate mediated excitotoxicity is one of the essential pathogenic factors involved in various neurodegenerative pathologies including AD (Goncalves-Ribeiro et al., 2019), neuroprotective effects of CEF were studied in AD models as well (Smaga et al., 2020; Kumari and Deshmukh, 2021).

The beneficial effects of CEF on AD-related pathology were revealed earlier using transgenic mouse AD models [3xTg-AD (Zumkehr et al., 2015) and APPPS1 (Hefendehl et al., 2016) strains] or a genetic rat model of spontaneous AD (OXYS strain) (Tikhonova et al., 2017). In transgenic murine models of advanced stages of AD-like pathology with highly expressed Aβ plaques (Zumkehr et al., 2015; Hefendehl et al., 2016), CEF neuroprotective effects were attributed to the attenuation of glutamatergic excitotoxicity induced by Aβ deposits, while no pronounced effect on APP processing, overall Aβ species levels (except for the increase in Aβ40 levels in the CEF-treated mice), or plaque pathology was observed (Zumkehr et al., 2015). In 5-month-old OXYS rats that correspond to an early stage of AD-like progression, our group revealed novel targets of CEF as it modulated the expression of genes related to the system of Aβ metabolism in the brain, namely, it affected mRNA levels of *Bace1*, *Ace2*, *Mme*, *Ide*, *Ece1*, and *Epo* (Tikhonova et al., 2018). Here, we checked whether CEF might influence Aβ burden at early stages of AD-like pathology. Indeed, in mice subjected to central administration of the Aβ25–35, Aβ deposition was significantly reduced after CEF therapy in the frontal cortex and hippocampus. Thus, we confirmed the CEF effects on Aβ-related hub of AD-like pathology. One may suggest that in the models of advanced stages of AD-like pathology with highly expressed Aβ deposits, those mechanisms activating enzymes of Aβ degradation are insufficient for considerable clearance from Aβ aggregates. In these cases, activation of other mechanisms such as macroautophagy that are responsible for segregation and eradication of pathological protein aggregates appears to be of benefit (Xin et al., 2018). It should be noted that CEF does not induce autophagy but rather has an inhibitory effect (Cui et al., 2014; Korolenko et al., 2020). In the mouse Aβ-induced AD model, CEF treatment reduced the augmented autophagy level in the brain (Korolenko et al., 2019).

Another process contributing much to the AD pathology is neuroinflammation. It is considered to be tightly involved into the amyloid cascade (Selkoe and Hardy, 2016). Hence, one may expect attenuation of Aβ-induced neuroinflammation due to the reduction of Aβ burden after CEF treatment. Indeed, the expression of a proinflammatory marker CD54 was substantially reduced by CEF in both the frontal cortex and hippocampus. However, the expression of a marker of microglia activation IBA1 was decreased in the frontal cortex after CEF treatment, but it remained augmented in the hippocampal regions. Moreover, no significant correlation was found between Aβ accumulation and the neuroinflammatory markers in the certain hippocampal areas. We consider that more complicated mechanism of the CEF anti-inflammatory effect takes place. Besides Aβ-related effect, effects of the CEF on other pathways regulating and modulating microglia function might be proposed. The suggestion is in a good agreement with recent findings on the CEF effects on microglial phagocytosis of glutamatergic synapses in the hippocampus of rats microinjected with Aβ1–40 through the reduction of synaptic production of the complement C1q (Wu et al., 2020).

AD has a multifactorial etiology and involves various pathological processes (e.g., neurotoxicity of protein aggregates, oxidative stress, neuroinflammatory response, disturbed neurotrophic function and neurogenesis, synaptic and neurotransmission dysfunction, ion disbalance, etc.) that often closely interact and overlap. Hence, multipurpose or multi-target therapy aimed at various important pathogenetic hubs in the course of AD is regarded currently as a relevant and promising approach (Sahoo et al., 2018). CEF appears to be a prospective drug of that kind as it potently and simultaneously targets glutamate excitotoxicity (Rothstein et al., 2005), oxidative pathways (Lewerenz et al., 2009; Stennett et al., 2017), neurotrophic function (Kaur and Prakash, 2017), neurogenesis (Ho et al., 2019), Aβ accumulation, and neuroinflammatory response as shown here.

Along with the beneficial effects on Aβ burden and neuroinflammatory response in the brain, CEF effectively prevented cognitive deficits in Aβ-treated mice. Aβ25–35 fragment used in the work is characterized by high neurotoxicity due to the high aggregative properties (Haass and Selkoe, 2007; Walsh and Selkoe, 2007). Although pharmacological Aβ-induced model of AD corresponds to early stages of AD-like pathology progression, mice or rats with Aβ-induced neurotoxicity demonstrate certain alterations in cognitive function including deficits in working memory, learning, or spatial memory (Park et al., 2011; Choi et al., 2013; El Bitar et al., 2014; Wu et al., 2020). In the present study, fear-associated memory and learning was considerably disturbed in Aβ-treated mice according to the passive avoidance test that is in a good agreement with a previous finding (Maurice et al., 1996). The behavioral response in the passive avoidance test was completely recovered by the CEF treatment. At the same time, the indices of working memory in the *T*-maze test or IntelliCage (patrolling behavior) or long-term spatial memory in the Barnes test were not significantly affected by Aβ administration. However, learning was slightly retarded in the Aβ25–35-treated groups on the first day of learning in the Barnes test or on the first day of the test for patrolling behavior in the IntelliCage; mice treated with Aβ25–35 revealed a retarded extinction of avoidance learning. Mice given CEF gained better scores when performing in the *T*-maze test (the working memory estimated) or in the Barnes test (long-term spatial memory and learning estimated) than the Aβ25–35 + saline group. In the IntelliCage, mice demonstrated different disturbances depending on a model of AD applied (Codita et al., 2010; Platt et al., 2011; Sekiguchi et al., 2011; Masuda et al., 2016). In the present study, we revealed a deficit of place learning on the first day of testing in the Aβ-treated mice that was reversed by the CEF treatment. It should be mentioned that the open field test revealed no significant differences in the indices of locomotion, exploratory activity, or anxiety between the controls and Aβ-treated mice or between the Aβ25–35 + saline and Aβ25–35 + CEF groups. Hence, the observed effect of CEF on cognitive function was specific and did not depend on general changes in locomotor or exploratory behavior. The beneficial effect of CEF on cognitive functions agrees well with the previous findings on the restoration of impaired cognition in the animal models of neurodegenerative disorders (Zumkehr et al., 2015; Weng et al., 2016; Tikhonova et al., 2017; Ho et al., 2019).

We may conclude that the CEF recovered Aβ-induced pathology and related cognitive impairment. Its neuroprotective activity involved the effects on Aβ burden and neuroinflammatory response in the brain. Hence, the CEF could be positioned as a potent multipurpose drug as it simultaneously targets proteostasis network and neuroinflammation, as well as glutamate excitotoxicity, oxidative pathways, neurotrophic function, and neurogenesis as reported earlier. Together with previous reports on the beneficial effects of the CEF in AD models (Zumkehr et al., 2015; Hefendehl et al., 2016; Tikhonova et al., 2017), the results of the study confirm the potential of the CEF as a promising treatment against cognitive decline from the early stages of AD progression.

## Data Availability Statement

The raw data supporting the conclusions of this article will be made available by the authors, without undue reservation.

## Ethics Statement

The animal study was reviewed and approved by Local Ethics Committee of the Scientific Research Institute of Neurosciences and Medicine.

## Author Contributions

MAT, TA, Y-JH, and LA: conceptualization. MVT, AA, MO, AB, and ND: methodology and formal analysis. MVT, AA, MO, and ND: investigation. MAT: data curation and writing—original draft preparation. MAT, MO, and AA: visualization. TA: writing—review and editing. MAT, TA, and LA: supervision, project administration, and funding acquisition. All authors have read and agreed to the published version of the manuscript.

## Conflict of Interest

The authors declare that the research was conducted in the absence of any commercial or financial relationships that could be construed as a potential conflict of interest.

## Publisher’s Note

All claims expressed in this article are solely those of the authors and do not necessarily represent those of their affiliated organizations, or those of the publisher, the editors and the reviewers. Any product that may be evaluated in this article, or claim that may be made by its manufacturer, is not guaranteed or endorsed by the publisher.

## Footnotes

1
https://clinicaltrials.gov/ct2/show/NCT03413384
