# Supplementary material for: Neuroprotective Effects of Ceftriaxone Involve the Reduction of Aβ Burden and Neuroinflammatory Response in a Mouse Model of Alzheimer’s Disease
Source: Front Neurosci. 2021 Sep 29;15:736786. doi: 10.3389/fnins.2021.736786 (PMC8511453; doi:10.3389/fnins.2021.736786)
Supplement: Supplementary file 1 [file Data_Sheet_1.docx]

**Table S1.** The IntelliCage protocol.

| ***Stage / test*** | ***Description*** | ***Duration, days*** | ***Days of the experiment*** |
| --- | --- | --- | --- |
| Free adaptation or habituation | Mice had free access to all four corners with water, all doors were open, water and food were available *ad libitum.* | 4 | (prior a surgery) |
| Nosepoke adaptation | All the doors were closed, and a mouse got access to water nosepoking at a door at any of the four corners. | 3 | (prior a surgery) |
| Stereotaxic surgery | Aβ or H_2_O administration into the lateral ventricles. | 1 | Day 0 |
| Free adaptation or habituation-2 | Mice had free access to all four corners with water, all doors were open, water and food were available *ad libitum.* | 3 | Days 1-3 |
| Nosepoke adaptation-2 | All the doors were closed, and a mouse got access to water nosepoking at a door at any of the four corners. | 3 | Days 4-6 |
| Place learning test | Each mouse had access to only one corner (“correct corner”) in which it can get water. The least preferred corner on the last day of adaptation was chosen as the “correct” one. The percentage of correct visits (visits to a “correct corner”) was recorded. | 5 | Days 7-11 |
| Place learning reversal test | A corner regarded as the “correct corner” was changed to an opposite one. The percentage of correct and incorrect visits was recorded. | 5 | Days 12-16 |
| Avoidance conditioning test | A mouse learned to avoid the corner where it is subjected to aversive punishment (an air-puff as an unconditioned stimulus) while drinking. For each mouse, one corner was designated as the “incorrect corner”, in which a nosepoke caused an air puff (0.8 bar, 1 s) until the animal leaved the corner and the door closed. The percentage of incorrect visits (visits to an “incorrect corner”) was recorded. | 3 | Days 17-19 |
| Avoidance extinction | Mice had been placed into home cage for one day (Day 20) with water restriction for 18 h prior return to IntelliCage. No aversive stimulus was used. A mouse could get access to water nosepoking at a door at any of the four corners. Avoidance extinction was assessed by the visits to a corner designated as an “incorrect” during the Avoidance conditioning test. The percentage of visits to the corner was recorded. | 5 | Days 21-25 |
| The test for patrolling behavior | The procedure for this test consisted of the alternation of corners with access to water. Mice had to move from one corner to the next in a clockwise direction in order to get access to water. The next rewarded corner was always adjacent to the corner most recently rewarded. The procedure started with the most preferred corner during the previous 24 h. When a mouse selected a “correct corner”, an additional signal was added (yellow LED was turned on); then a next corner became the “correct” one. Cognitive activity was estimated by the percentage of visits to the active rewarded corner. | 3 | Days 26-28 |

**Table S2.** Effects of the CEF and Aβ25-35 administration (AD model) on the neuronal density (according to Nissl staining) in the frontal cortex and hippocampus in mice.

| **Brain structure** | **Group** | | | | **Effects**  *(F, p)* |
| --- | --- | --- | --- | --- | --- |
|  | ***H_2_O+Saline*** | ***H_2_O+CEF*** | ***Aβ25-35+***  ***Saline*** | ***Aβ25-35+***  ***CEF*** |  |
| Frontal cortex | 30.2±1.0 | 29.8±1.0 | 29.3±1.9 | 30.4±1.4 | **Aβ:** *F*(1,8) < 1;  **CEF:** *F*(1,8) < 1;  **Aβ х CEF:** *F*(1,8) < 1 |
| CA1 | 15.6±2.8 | 13.8±1.7 | 13.7±1.2 | 15.2±1.6 | **Aβ:** *F*(1,8) < 1;  **CEF:** *F*(1,8) < 1;  **Aβ х CEF:** *F*(1,8) < 1 |
| CA3 | 14.8±0.8 | 15.2±0.4 | 13.9±0.6 | 14.2±1.0 | **Aβ:** *F*(1,8) < 1;  **CEF:** *F*(1,8) < 1;  **Aβ х CEF:** *F*(1,8) < 1 |

The data are expressed as the means ± SEMs of the values obtained in an independent group of animals (n=3 per group).
